# Capturing Parents’ Perspectives of Child Wellness to Support Identification of Acutely Unwell Children in the Emergency Department

**DOI:** 10.1097/PTS.0000000000000949

**Published:** 2021-12-17

**Authors:** Abigail Albutt, Damian Roland, Rebecca Lawton, Mark Conner, Jane O’Hara

**Affiliations:** From the ∗ Yorkshire Quality and Safety Research Group, Bradford Institute for Health Research, Bradford Royal Infirmary, Bradford; † Paediatric Emergency Medicine Leicester Academic (PEMLA) Group, Children’s Emergency Department, Leicester Royal Infirmary, Leicester; ‡ SAPPHIRE Group, University of Leicester, Leicester; § School of Psychology, University of Leeds, Leeds; ∥ School of Healthcare, University of Leeds, Leeds, United Kingdom.

**Keywords:** pediatric emergency department, patient deterioration, patient and family involvement

## Abstract

**Objective:**

Early signs of serious illness can be difficult to recognize in children and a delayed response can result in poor outcomes. Drawing on the unique knowledge of parents and carers may improve identification of the deteriorating child. However, there is a lack of evidence exploring parental perspectives as part of track and trigger systems. This study examines the utility of capturing parent-reported child wellness, using the Patient Wellness Questionnaire for Pediatrics, to support identification of acutely unwell children presenting to the Emergency Department.

**Methods:**

Parent-reported child wellness was recorded alongside the Pediatric Observation Priority Score (POPS), a multidimensional scoring system akin to a Pediatric Early Warning Score, used as part of routine care. Multiple linear regression assessed the independent effects of 3 variables (parent-reported child wellness, nurse concern, and child age) on POPS and hospital admission.

**Results:**

Data were collected for 407 children. All 3 variables explained a statistically significant proportion of variance in POPS (*F*(3, 403) = 7.525, *P* < 0.001, *R*^2^ = 0.053), with parent-reported child wellness (*B* = 0.223, SE = 0.054, β = 0.202, *P* < 0.001) having the strongest effect. Approximately 10% of the children with no physiological derangement were rated as “very poorly” by their parents.

**Conclusions:**

The findings suggest that parents have insight in to the wellness of their children that is reflected in the physiological assessment. Some parents’ perceptions about their child’s wellness were not consistent with the score captured in the same assessment. More work is needed to understand how to use and address parental perspectives and concerns to support clinical decision making and the management of acute illness.

Managing clinical deterioration in hospital remains a global problem, and deterioration in some patients is not promptly recognized, or responded to, resulting in adverse outcomes.^1,2^ Measures are in place to support the early detection and escalation of patient deterioration in hospital, for instance, track and trigger systems, sometimes referred to as early warning scores. These involve the observation of physiological signs with predefined response criteria that trigger support from staff with critical care skills if patients are deteriorating after intervention from the ward team.^3^

It is increasingly recognized that patients can make important contributions toward their own safety in hospital and initiatives have encouraged patient involvement in patient safety.^4,5^ The Patient Wellness Questionnaire (PWQ) was developed by researchers to support healthcare staff to ask hospitalized adult patients about changes in their health and wellness.^6,7^ Subsequent studies found an association between patients’ perceptions of their wellness captured using the PWQ and objective measures of their physiology (National Early Warning Score).^8^ Importantly, this research also suggested that patients may identify signs of deterioration before their physiological measurements demonstrate this.^7^

It can be difficult to recognize serious illness in children and early signs of deterioration may not be noticeable.^9^ This can pose a challenge in emergency and urgent care departments, where large numbers of children attend, but only a small proportion are seriously unwell.^9,10^ Anecdotal evidence suggests that parents and carers may be well placed to recognize early signs and symptoms of deterioration in their child’s condition^11–13^ and drawing on this could improve identification of the deteriorating child.^14,15^ However, parental concern measures are only being to be used in a small number of pediatric track and trigger systems in the United Kingdom.^15,16^ The Pediatric Observation Priority Score (POPS)^9^ is one such approach that measures vital signs and includes subjective observational criteria, such as nurse concern and nurse perceptions’ of parental concern. It has been specifically designed to not only highlight patients of higher acuity but also assist in safe discharge decisions for children of low acuity.

The PWQ, previously described, could offer a powerful means (if adapted) to capture parents’ views of their child’s wellness in emergency and acute care settings. This study reports on such a measure, adapted in collaboration with parents and pediatric health professionals and referred to as the Patient Wellness Questionnaire for Pediatrics (PWQP). This study aims to explore whether using the PWQP to directly measure parental perceptions of their child’s wellness, alongside an emergency department (ED) scoring system (POPS), adds to the identification of acutely unwell children. This exploratory study addresses the following questions: (1) Are parent-reported child wellness and nurse concern associated with POPS? (2) Are parent-reported child wellness, nurse concern and POPS associated with admission to hospital?

## METHOD

### Setting and Participants

This study was conducted at a pediatric ED within a large teaching hospital in England. The data were collected in January to February 2020.

### Measures

#### Patient Wellness Questionnaire for Pediatrics

The PWQ, developed in a previous study,^6^ can be used by healthcare staff to ask hospitalized adult patients about changes in their health and wellness. The assessment includes 2 questions each with the following 5 response options: question 1: How are you feeling? Very poor (5), poor (4), fair (3), good (2), very good (1), and question 2: How are you feeling compared with the last time you were asked? Much worse (5), worse (4), no change (3), better (2), and much better (1).

Consultation meetings were conducted first with 5 individual pediatric health professionals (head of nursing for children’s services, advanced pediatric nurse practitioner, consultant pediatrician ×3) who were shown the PWQ and asked how it could be adapted for use with parents and children. Suggestions from the health professionals were then discussed with 6 parents during a parent governors meeting. Comments and suggestions from pediatric health professionals and parents were collated using feedback forms, and a further meeting was held with the research team to amend the PWQ to create the PWQP. The wording and utility of questions and response options in the PWQP were also discussed with nurses during interviews in the current study that will be reported elsewhere. The PWQP questions and response options are as follows: question 1: How do you think your child is feeling? Very poor (1), poor (2), fair (3), good (4), very good (5), and Question 2: How do you think your child is feeling compared to the last time you were asked? Much better (1), better (2), no change (3), worse (4), much worse (5). Where the child has capacity and is of suitable age to give a response, they can be asked the PWQ.

#### Pediatric Observation Priority Score

The POPS is a form of early warning score, specific to children, which includes the traditional vital sign measurements of heart rate, temperature, respiratory rate, and oxygen saturations. Additional objective criteria (alert, voice, pain, unresponsive scale and medical history) and subjective observational criteria (work of breathing and nurse concern) are added to create POPS.^9^ It is scored 0 to 16 with a score of 8 and greater meaning that the child should be considered for immediate care. The POPS has been internally^9^ and externally validated^17^ and is recommended by the intercollegiate committee for standards for children and young people in emergency care settings.^18^ Scoring systems used in children’s emergency care have traditionally been poorly predictive of admission in a reliable way,^19^ yet there is a clear association between POPS and admission (the higher the POPS the more likely that admission occurs ROC 0.802).^20^ The POPS was designed as a cognitive prompt to aid decision making rather than delineate pathways of clinical care.

### Procedure

The study was conducted as part of routine care during the study period. Patients were booked into the ED at the participating hospital and had an assessment within 15 minutes of arriving in the department (current departmental standard), which included a POPS score recorded by the triage or assessment nurse on duty. For all children who got a POPS at the assessment, the nurse also recorded a PWQP score. This information was recorded in the free text area of the local electronic health record. The study population was children younger than 16 years. Nursing staff used their clinical judgment and information about the age and capacity of the child to determine whether they asked the parent to give a response to PWQP or the child themselves.

An opt-out approach to parent and child recruitment was used. Nursing staff outlined how parents and children would be involved in the study during the assessment and gave them the opt-out consent form. If parents did not want their children’s data to be used in the study, they could sign the opt-out form and leave it at the registration desk. For parents who opted out, data were identified by using the date of their visit to the ED and their waiting number. The only data collected that would not already be obtained from patients was the PWQP scores. The data (POPS, PWQP scores, and nonidentifying patient demographics such as age) can be extracted anonymously from the secure encrypted data base that contains the electronic patient record. Data collection was conducted for 8 weeks. The POPS and PWQP scores and decision to admit to hospital were collected for children during ED assessments.

### Data Analysis

The analysis explored the association between parent-reported child wellness (assessed using the PWQP), level of nurse concern, age, and POPS recorded during the same assessment of the child. The PWQP scores were reversed, and the nurse concern score was removed from POPS for all patients in the analysis. Multiple linear regression was also conducted to explore the independent effect of 3 variables (age, PWQP, and nurse concern) on POPS and admission to hospital. A more detailed analysis of the relationship between PWQP scores and POPS was conducted via cross-tabulation of scores.

### Ethical Approval

Ethical approval for this study was granted by the National Health Service Health Research Authority London Bridge Research Ethics Committee (Reference: 19/HRA/4721).

## RESULTS

Data were collected for 407 children and 5 parents opted out of the study before data collection. Reasons for opting out were not given. Table 1 reports descriptive statistics and the zero-order (Pearson) correlations between measured variables. In the cohort of recruited participants, the admission rate was 17.2% (70 of 407). In the period of the study, the admission rate to the ward or short stay unit for all presentations was 17.9% (850 of 4746). The PWQP was the strongest correlate of POPS (*r*(405) = 0.19, *P* < 0.01), although the effect size was small. Age (negative) was also significantly correlated with POPS (*r*(405) = −0.11, *P* < 0.05) and the magnitude of effect was small. Nurse concern was not significantly correlated with POPS. The PWQP, age, and nurse concern were not found to be significantly correlated with admission to hospital. The POPS was found to be significantly positively correlated with admission to hospital with a small-medium effect size (*r*(405) = 0.28, *P* < 0.01).

**TABLE 1:** Descriptive Statistics and Person’s Correlation Matrix for Measured Variables (*N* = 407)

	Mean	Standard Deviation	PWQP	Nurse Concern	POPS	Admission to Hospital
Age	4.08	4.38	0.090	0.005	−0.111*	0.038
PWQP	2.69	1.15		−0.029	0.191†	0.081
Nurse concern	0.04	0.20			−0.015	0.043
POPS	1.10	1.27				0.283†
Admission to hospital	1.18	0.38				

*P < 0.05.

†*P* < 0.01.

Multiple linear regression was used to assess the independent effects of the 3 variables (PWQP, nurse concern, and age) on POPS (Table 2). Together, these 3 variables explained a small but statistically significant proportion of variance in POPS (*F*(3, 403) = 7.525, *P* < 0.001, *R*^2^ = 0.053). Two variables were significant with PWQP (*B* = 0.223, SE = 0.054, β = 0.202, *P* < 0.001) having the strongest effect, followed by age (*B* = −0.037, SE = 0.014, β = −0.129, *P* < 0.01). Nurse concern was not significantly associated with POPS (*B* = −0.053, SE = 0.305, β = −0.008, *P* = 0.861).

**TABLE 2:** Summary of Hierarchal Regression Analysis for Variables Predicting POPS (*N* = 407)

	Model 1			Model 2			Model 3		
Variable	*B*	SE *B*	β	*B*	SE *B*	β	*B*	SE *B*	β
PWQP	0.211	0.054	0.191*	0.210	0.054	0.191*	0.223	0.054	0.202*
Nurse concern				−0.059	0.308	−0.009	−0.053	0.305	−0.008
Age							−0.037	0.014	−0.129†

Model 1: *ΔR*^2^ = 0.036, *ΔF*(1, 405) = 15.32, *P* < 0.001; model 2: *ΔR*^2^ = 0.000, *ΔF*(1, 404) = 0.04, *P* = 0.847; model 3: *ΔR*^2^ = 0.017, *ΔF*(1, 403) = 7.03, *P* = 0.008.

*P < 0.001.

†*P* < 0.01.

Cross-tabulation of PWQP scores by the POPS indicated that 11.3% (19 of 168) of the children with a POPS of 0 were rated as “very poorly” by their parents (Table 3). Of these 19 children, 2 were admitted to a short stay unit and subsequently discharged. One child in the sample had a POPS of 7 indicating high acuity and was rated as “good” by their parents. Binary logistic regression indicates that POPS was a significant predictor of hospital admission (*B* = 0.541, SE = 0.103, *P* < 0.001). The PWQP (*B* = 0.069, SE = 0.127, *P* = 0.584), age (*B* = 0.049, SE = 0.031, *P* = 0.149), and nurse concern (*B* = 0.189, SE = 0.646, *P* = 0.352) were not significant predictors of admission to hospital (Table 4).

**TABLE 3:** Cross-Tabulation of PWQP Scores by POPS

PWQP						
POPS	Very Poorly	Poorly	Fair	Good	Very Good	Totals
0	19	30	49	54	16	168
1	14	19	26	39	19	117
2	3	4	20	32	9	68
3	1	2	6	17	7	33
4	0	3	1	6	1	11
5	0	1	2	3	2	8
6	0	0	0	1	0	1
7	0	0	0	1	0	1
Totals	37	59	104	153	54	407

**TABLE 4:** Summary of Hierarchal Regression Analysis for Variables Predicting Admission to Hospital (*N* = 407)

Variable	*B*	SE *B*	OR	95% CI OR
Model 1				
PWQP	0.193	0.120	1.213	0.959–1.536
Nurse concern	0.192	0.618	1.212	0.361–4.074
Age	0.021	0.029	1.021	0.964–1.081
Model 2				
PWQP	0.069	0.127	1.072	0.835–1.376
Nurse concern	0.189	0.646	1.208	0.340–4.289
Age	0.049	0.031	1.050	0.988–1.117
POPS	0.541	0.103	1.717*	1.404–2.100

Model 1: *Δ*Nagelkerke *R*^2^ = 0.014, *Δ*χ^2^ = 3.46, *P* = 0.326; model 2: *Δ*Nagelkerke *R*^2^ = 0.116, *Δ*χ^2^ = 29.72, *P* < 0.001.

*P < 0.001.

CI, odds ratio; OR, odds ratio.

## DISCUSSION

In this study, nurses were invited to record parent-reported child wellness using the PWQP during initial assessments when children presented to the ED. The study sought to examine the association between parent-reported child wellness captured using PWQP and POPS and to understand whether parental concern captured using PWQP can add to the identification of deteriorating children. The findings provide insight in to the clinical utility of parents’ responses to the PWQP.

The PWQP was most strongly correlated with POPS (*r = 0*.191, *P* < 0.01) indicating that when children were more unwell according to clinical observations (higher acuity), parents reported their children to be increasingly unwell. This suggests that parents have insight into the wellness of their children that is reflected in the physiological assessment within POPS. Approximately 10% of children with the lowest POPS, indicating no physical derangement, were perceived to be “very poorly” by their parents. In these cases, there seemed to be a disconnect between parents’ views of their child’s wellness and objective vital signs measured. There are 2 possible explanations for this, both of which might be at play in different situations. First, the findings suggest that there may be limits to the usefulness of recording parent-reported wellness in this setting. Perhaps some parents in some situations are more anxious than they need to be. Conversely, of course, it may be that parents are recognizing subtle signs of illness before they are highlighted in physiological measures. Parents may be able to anticipate deterioration. The data here do not distinguish between these 2 explanations. However, where this disconnect is apparent further exploration of the reasons for this from a parental perspective is needed, and appropriate interventions (reassurance of parents and safety netting, for example) may be necessary. Further funding is being sought, and there are plans to explore this in a future study.

The POPS was significantly associated with admission to hospital, which is expected as it is a criteria used to support triage and subsequent decisions to admit children to hospital. However, PWQP and nurse concern were not significant predictors of the decision to admit. This suggests that health professionals use scoring systems, and other unmeasured variables, to underpin their decision to admit a child. In some instances, nurse concern may help ensure that a child is prioritized for medication or pain relief to allow the child to be discharged rather than admitted. It is known, however, that health professionals may overlook vital information from parents. The following specific cases demonstrate instances of parents’ recognizing early, subtle signs that their child was deteriorating, for example, in the cases of 18-month-old Josie King^11^ and 15-year-old Lewis Blackman.^13^ In these cases, there was not a timely response or escalation of care resulting in the unexpected death of these children.

Such high profile cases led some hospitals to introduce family activated rapid response teams, allowing parents to call for help if they feel their child is deteriorating and requires attention from staff with critical care skills.^11,21^ Research evaluating these systems suggests that a high number of calls to rapid response teams involve nonsafety-related issues.^22,23^ It is plausible that using the PWQP may enable parents to tell staff about changes in their child’s wellness before acute deterioration in a sequential fashion. Recording parent-reported child wellness alongside objective vital sign measurements may support staff decision making about admission to hospital and improve management of deterioration. This should be explored in a ward based study of PWQP and an inpatient pediatric early warning scoring system.

A limitation of this study was that based on their scores when presenting to the ED, most children in the sample were generally well. As such, parent-reported wellness was collected for children who were mostly well enough to receive primary care or go home. The findings may have been different if a greater number of acutely unwell children presented to the ED during the study period. For instance, previous research in an adult inpatient setting highlighted a stronger association between patient-reported wellness and subsequent vital sign measurements for patients who were more acutely unwell.^7^ It was the intention for nurses to note whether the parent or child responded to the PWQP, but because of the difficulty of introducing new processes in the study within an extremely busy ED, it was not known whether parents or older children responded to the PWQP. The second item within the PWQP intended for use during follow-up observations asks “How do you think your child is feeling compared to the last time you were asked?” A cross-sectional design was used, and therefore, this follow-up item was not used in the study because decisions are often made in EDs on the basis of only 1 or very few observations.^24^ Future studies should assess the utility of the follow-up question within PWQP.

Further larger-scale research exploring parent-reported wellness, physiological measurements, and outcomes for patients, who are admitted, or who may represent to the ED is also needed. This would shed light on the possibility that parents may recognize signs and symptoms that preempt acute illness and whether acting on parental perspectives of wellness could prevent further decline. This study provides the first step in a larger program of research and a means for raising awareness within the scientific community that this is a topic that requires further and rigorous exploration.

## CONCLUSIONS

The findings show that parent-reported wellness captured using the PWQP was significantly associated with POPS, a scoring system using physiological vital signs. Some parents’ perceptions about their child’s wellness were not consistent with the score captured in the same assessment. It may be that these responses to the PWQP are not clinically useful, or it could be that parents are tapping in to subtle changes in their child’s wellness that are not yet shown in physiological vital signs, giving an opportunity for early recognition and response. More work is needed to understand how to use parental perspectives and concerns regarding their child’s condition to support clinical decision making.
